# A Rare Case of Platypnea-Orthodeoxia Syndrome in a Patient With Undiagnosed Atrial Septal Defect

**DOI:** 10.7759/cureus.61260

**Published:** 2024-05-28

**Authors:** Rei Sanai, Tetsuo Hirata, Toyoshi Yanagihara, Meimi Okamoto, Takato Ikeda, Yuki Shundo, Naoki Hamada, Noriyuki Ebi, Hiroyuki Inoue, Shin-ichiro Miura, Masaki Fujita

**Affiliations:** 1 Department of Respiratory Medicine, Fukuoka University Hospital, Fukuoka, JPN; 2 Department of Cardiology, Fukuoka University School of Medicine, Fukuoka, JPN

**Keywords:** microbubble test, right-to-left shunt, dyspnea, atrial septal defect, platypnea-orthodeoxia syndrome

## Abstract

Platypnea-orthodeoxia syndrome (POS) is a rare condition characterized by dyspnea and oxygen desaturation that worsens in the upright position and improves when lying down. We report the case of a 67-year-old male who presented with a 14-month history of dyspnea in the sitting/standing position. Despite treatment for suspected asthma, his symptoms persisted, and he was referred to our hospital for further evaluation. Physical examination and arterial blood gas analysis confirmed the presence of POS, with a significant decrease in PaO_2_ and SpO_2_ when moving from a supine to an upright position. Contrast-enhanced CT showed no obvious embolism nor arteriovenous fistula, and ventilation-perfusion scintigraphy demonstrated ventilation-perfusion mismatch with a right-to-left shunt fraction of 9.4%, without any focal defect. Transthoracic echocardiography with a microbubble test demonstrated a right-to-left shunt that increased in the upright position. Transesophageal echocardiography revealed an atrial septal defect (ASD) with an atrial septal aneurysm and the presence of an inferior vena cava valve, causing a bidirectional shunt. The patient was diagnosed with POS secondary to ASD and was referred for percutaneous closure of the defect. Following the procedure, the shunt resolved, and the patient’s orthostatic oxygen desaturation improved. This case highlights the importance of considering POS in patients with positional dyspnea and the value of performing diagnostic tests, such as echocardiography, in different positions to identify the underlying cause. Early recognition and appropriate management of POS can significantly improve patients’ quality of life and prevent complications associated with chronic hypoxemia.

## Introduction

Platypnea-orthodeoxia syndrome (POS) is a rare condition first described by Burchell [1], characterized by dyspnea and hypoxemia that worsens in the upright position and improves when lying down. The pathophysiology of POS can be attributed to two main mechanisms: intracardiac shunting and ventilation-perfusion mismatch [2]. Intracardiac shunting, typically caused by atrial septal defects (ASDs) or patent foramen ovale (PFO), can lead to POS when combined with elevated right atrial pressure or anatomical changes. Conditions such as pulmonary embolism, pulmonary arterial hypertension, aortic elongation, or kyphoscoliosis can contribute to the development of POS in the presence of an intracardiac shunt. Alternatively, POS can result from ventilation-perfusion mismatch in the setting of pulmonary diseases such as hepatopulmonary syndrome or interstitial lung disease. In these cases, the underlying pulmonary pathology leads to an uneven distribution of ventilation and perfusion, resulting in hypoxemia that worsens in the upright position.

Due to its rarity and varied etiologies, the diagnosis of POS can be challenging. In this report, we present a case of POS secondary to ASD, highlighting the diagnostic difficulties encountered and the importance of considering this syndrome in patients presenting with positional dyspnea and hypoxemia.

## Case presentation

A 67-year-old male presented with a 14-month history of dyspnea. He reported that his breathlessness worsened when sitting or standing, but paradoxically improved when walking. During episodes of severe dyspnea, he found relief by lying down. His symptoms persisted despite oxygen therapy. Eight months before presentation, he was clinically diagnosed with asthma at another hospital and treated with inhaled medications, but his symptoms did not improve. Although his oxygenation was adequate in the supine position, his SpO_2_ (oxygen saturation as measured by pulse oximeter) decreased to 70% when sitting, and platypnea was suspected. Four months before presentation, the patient underwent contrast-enhanced CT, transthoracic echocardiography, and cardiac catheterization at the previous hospital, but the cause of his symptoms was not identified. He was referred to our hospital for further evaluation. The patient’s medical history included hypertension. He was a past smoker with a 46-year history of smoking 20 cigarettes per day until age 66 and was an occasional drinker. His family history was unremarkable.

On examination, his vital signs were as follows: pulse rate 80 beats/minute (regular), blood pressure 116/86 mmHg, and SpO_2_ 98% in the supine position, decreasing to 75% when sitting (room air). His height was 163 cm and his weight was 65.7 kg. Cardiac auscultation revealed normal S1 and S2 without murmurs or split S2. Lung sounds were normal without adventitious sounds. There was no jugular venous distension or peripheral edema. His SpO_2_ raised up to 92% after the six-minute walk test.

**Figure 1:**
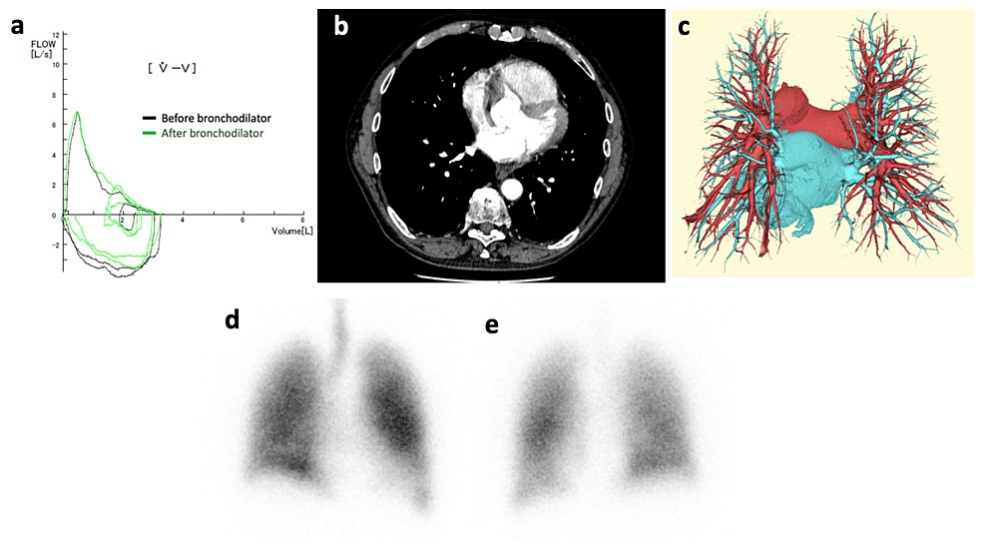
Pulmonary function, CT, and scintigraphy findings of the patient. (a) The flow-volume curve of the patient shows a downward convex. X-axis: volume (L), Y-axis: flow (L/second). (b) An axial enhanced CT image of the chest shows no obvious pulmonary artery embolism. (c) Three-dimensional reconstructed contrast-enhanced CT images of the pulmonary vasculature, with red vessels representing the pulmonary arteries and blue vessels representing the pulmonary veins. No arteriovenous fistula is observed. (d) Ventilation and (e) perfusion scintigraphy show no focal defect but ventilation-perfusion mismatch with a right-to-left shunt fraction of 9.4%.

Arterial blood gas analysis showed PaO_2_ of 90.8 Torr, PaCO_2_ of 29.9 Torr, and A-aDO_2_ (alveolar-arterial oxygen difference) of 21.5 Torr in the supine position, while in the sitting position, PaO_2_ was 42.0 Torr, PaCO_2_ was 26.1 Torr, and A-aDO_2_ was 75.1 Torr, consistent with a diagnosis of POS. Blood tests showed a D-dimer of 0.6 µg/mL, brain natriuretic peptide of 17.0 pg/mL, and C-reactive protein of 0.14 mg/dL. Liver and kidney function tests and electrolytes were within normal limits (Table 1). Pulmonary function tests revealed mild obstructive impairment with a forced vital capacity of 3.57 L, forced expiratory volume in one second (FEV1) of 2.34 L, FEV1% of 65.5%, and %FEV1 of 82.4% (Figure 1a). Diffusion capacity could not be measured due to severe dyspnea. Contrast-enhanced CT showed no obvious embolism or arteriovenous fistula (Figures 1b, 1c). Ventilation-perfusion scintigraphy demonstrated ventilation-perfusion mismatch with a right-to-left shunt fraction of 9.4%, without any focal defect (Figures 1d, 1e).

**Table 1:** Serological examination of the patient on admission. WBC = white blood cell; RBC = red blood cell; AST = aspartate transaminase; ALT = alanine transaminase; LDH = lactate dehydrogenase; CK = creatine kinase; BUN = blood urea nitrogen; CRP = C-reactive protein; KL-6 = Krebs von den Lungen-6; BNP = brain natriuretic peptide; TSH = thyroid-stimulating hormone; PT = prothrombin time; APTT = activated partial thromboplastin time

Test	Value	Units
WBC	4,800	/μL
RBC	438	10^4^/μL
Hemoglobin	14.6	g/dL
Platelets	29.2	10^3^/μL
Total protein	7.3	g/dL
Total bilirubin	1.2	mg/dL
AST	16	U/L
ALT	15	U/L
LDH	165	U/L
CK	57	U/L
BUN	12	mg/dL
Creatinine	1.05	mg/dL
Na	144	mEq/L
K	3.8	mEq/L
CRP	0.14	mg/dL
KL-6	147	U/mL
BNP	17	pg/mL
Troponin I	<10.0	pg/mL
Free T4	1.31	ng/mL
TSH	1.89	mIU/L
PT	11.7	Seconds
APTT	30.2	Seconds
D-dimer	0.6	μg/mL

Transthoracic echocardiography showed a preserved ejection fraction (60.7%), normal left ventricular diastolic function, nearly normal right ventricular function, no significant valvular regurgitation, but with a slightly dilated aorta (37.6 mm) (Table 2). A microbubble test revealed a grade 2 shunt ﻿within three cardiac cycles in the supine position, increasing to grade 4 in the upright position (Video 1).

**Table 2:** Transthoracic echocardiogram of the patient. LAD = left atrial dimension; AOD = aortic dimension; LVDd = left ventricular diastolic diameter; LVDs = left ventricular systolic diameter; IVS = interventricular septum; LVPW = left ventricular posterior wall; EF = ejection fraction; FS = fractional shortening; TDI = tissue Doppler imaging; TMF = transmitral flow pattern; LV EDV = left ventricular end-diastolic volume; LV ESV = left ventricular end-systolic volume; SV = stroke volume; LVOT = left ventricular outflow tract; LAV = left atrial volume; TAPSE = tricuspid annular plane systolic excursion; FAC = fractional area change

Dimension	Value	Units	Reference	TMF	Value	Units	Reference
LAD	38.6	mm	19–40	E	66.7	cm/s	70–100
AOD	37.6	mm	20–37	A	80.3	cm/s	45–70
LVDd	39.2	mm	32–54	E/A	0.83	-	1.0–1.5
LVDs	26.7	mm	22–39	DcT	344	ms	160–240
IVS	9.4	mm	6–12	Volume	Value	Units	Reference
LV PW	9.2	mm	8–12	LV_EDV	57.4	mL	53–141
EF	60.7	%	50–88	LV_ESV	20.1	mL	13–69
FS	32	%	>25	EF	65	%	50–88
TDI	Value	Units	Reference	SV(LVOT)	86.6	mL	-
e' (septal)	4.6	cm/s	-	LA volume	62.1	mL	-
e' (lateral)	6.3	cm/s	-	LAV index	35.9	mL/m^2^	-
E/e' (sep)	14.5	-	-	Right ventricular function	Value	Units	Reference
-	-	-	-	TAPSE	16.2	mm	>17
-	-	-	-	FAC	55	%	>35

**Video 1:** Righ-to-left shunt revealed by echocardiography. (a) Transthoracic echocardiography with a four-chamber view of the heart at the spine position showing the presence of microbubbles in the left atrium and left ventricle within three cardiac cycles, suggesting a grade 2 right-to-left shunt. (b) The spine position on the examination bed. (c) Transthoracic echocardiography with a four-chamber view of the heart at the standing position showing the increase of microbubbles in the left atrium and left ventricle, suggesting a grade 4 right-to-left shunt. (d) The standing position on the examination bed.

**Figure 2:**
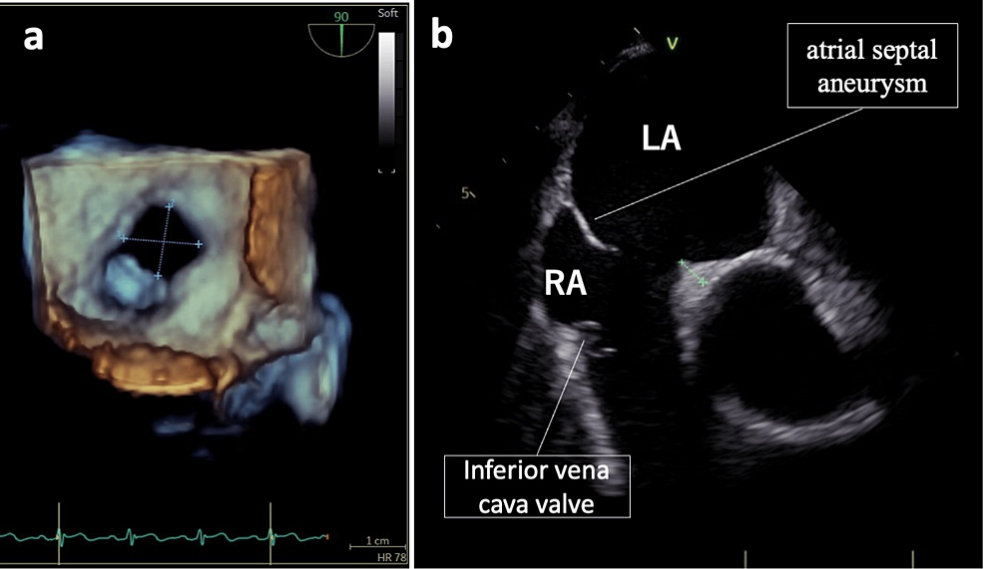
Atrial septal defect detected by transesophageal echocardiography. (a) Transesophageal echocardiography performed in the supine position reveals an atrial septal defect associated with an atrial septal aneurysm. The diameter of the ASD measured is 13 mm. (b). Additionally, an inferior vena cava valve is visualized. LA = left atrium; RA = right atrium

Transesophageal echocardiography in the supine position demonstrated ASD with an atrial septal aneurysm and an inferior vena cava valve (Figure 2). At rest, there was a left-to-right shunt, which became bidirectional with the Valsalva maneuver. The microbubble test showed a grade 1 shunt in the supine position, increasing to grade 3 with the Valsalva maneuver. To assess potential complications of the right-to-left shunt, such as paradoxical embolism, an MRI of the brain and CT images of the body were obtained. These imaging studies showed no evidence of thromboembolism.

Based on these findings, the patient was diagnosed with POS associated with ASD with an atrial septal aneurysm. As treatment was difficult at our hospital, the patient was referred to another hospital for percutaneous ASD closure. Right heart catheterization was performed, and the following data were obtained: right atrial pressure, 4 mmHg; mean pulmonary artery pressure, 17 mmHg; mean pulmonary capillary wedge pressure, 7 mmHg; oxygen saturation: superior vena cava, 73.5%, inferior vena cava, 67.7%, pulmonary artery, 84.7%; ASD defect size, 19 mm; pulmonary to systemic blood flow ratio, 1.9; and shunt ratio, 46%. The data suggested that no pulmonary hypertension existed. Following the procedure, echocardiography confirmed the disappearance of the shunt flow, and the patient’s orthostatic oxygen desaturation resolved with an SpO_2_ of 98% in the standing position.

## Discussion

POS is a rare condition first described by Burchell in 1949. Since then, approximately 200 cases have been reported up to 2016 [3]. POS is defined as a decrease in PaO_2_ of 4 Torr or more, or a decrease in SaO_2_ (arterial oxygen saturation) of 5% or more when moving from a supine to an upright position. In the present case, the patient experienced a decrease in PaO_2_ of 48 Torr and a decrease in SpO_2_ of 15%, meeting the diagnostic criteria for POS. The patient’s accompanying complaint that walking for a short while made him feel better is also suggestive of POS. This improvement with walking may be attributed to an increase in systemic vascular resistance during exercise, which leads to an elevation in left atrial pressure. Consequently, the increased left atrial pressure likely reduced the magnitude of the right-to-left shunt, thereby alleviating his symptoms. Indeed, the patient’s SpO_2_ after the six-minute walk test was higher than that in the sitting position at rest, supporting this hypothesis. The present case is worth reporting because the patient’s chief complaint was highly suggestive of POS, and the changes in SpO_2_ during different positions and activities provided strong evidence to support the diagnosis.

POS can be caused by either cardiac or pulmonary disorders. In cases of POS with a cardiac origin, two elements must coexist: an anatomical abnormality and a functional disturbance [4]. The anatomical component is characterized by the presence of a structural defect that permits communication between the atria, such as PFO, ASD, or a fenestrated atrial septal aneurysm. The functional component, on the other hand, refers to any condition that can cause a deformity of the atrial septum, resulting in the shunting of blood between the atria when the patient assumes an upright position [5].

While the exact prevalence varies across studies, cardiac abnormalities are the main cause of POS, and ASD emerges as one of the most common cardiac abnormalities leading to the development of POS, second to PFO [6]. We can speculate the frequency based on the prevalence of ASD and the reported cases of POS due to ASD. The estimated prevalence of ASD worldwide is around 56 per 100,000 live births [7]. Assuming the world population is approximately 8 billion, the estimated number of patients with ASD can be calculated as 448,000. ASDs are a common underlying cause of POS, with 18 cases specifically attributed to ASDs in a comprehensive review by Rodrigues et al. [6]. Since then, several additional cases have been reported, including three patients described by Takahashi et al. [8]. Based on the simple calculation, the estimated incidence of POS among patients with ASD would be 0.004%. However, the true prevalence may be underestimated due to the challenges in diagnosing POS and the potential for asymptomatic or mildly symptomatic ASDs to go undetected. Continued reporting and investigation of POS cases associated with ASDs are essential to better understand the true burden of this condition and improve patient care.

According to the literature, POS is typically diagnosed in older individuals, with the median age of onset being in the seventh decade of life [6]. This delayed presentation suggests that age-related factors may contribute to the development of POS in patients with congenital ASDs. Aortic dilatation, aneurysm, or distortion, observed in 23.4% to 63% of POS patients [6,9], can induce changes in the atrial septum, potentiating right-to-left shunts. Kyphoscoliosis and inferior vena cava valve may also contribute to altered blood flow patterns, exacerbating right-to-left shunting through an ASD [4,6,10]. In the upright position, the direction of venous return from the inferior vena cava shifts toward the atrial septum, facilitating the shunt from the right to the left atrium [7]. In the present case, the development of an atrial septal aneurysm and aortic dilatation during the aging process along with an ASD and an inferior vena cava valve may have played a crucial role in the onset of POS.

Various diagnostic modalities are used to diagnose POS, including transthoracic echocardiography, transesophageal echocardiography, contrast-enhanced CT, and MRI. While transthoracic echocardiography is non-invasive and readily available, its sensitivity is lower than that of transesophageal echocardiography. The sensitivity of transthoracic echocardiography for detecting ASD has been reported to be 50.0% [11]. Notably, in cases where the defect is small, and there is no right ventricular enlargement, the presence of ASD may not be suspected on cross-sectional imaging, making it difficult to identify the shunt flow. Nonetheless, transthoracic echocardiography has been reported to reveal a PFO when the patient’s position is changed, even if the shunt is not evident in the supine position [12]. Transesophageal echocardiography allows for a detailed evaluation of the atrial septum and quantification of the shunt [13,14]. Transesophageal echocardiography has also demonstrated an increase in shunting when the patient’s position is changed from supine to sitting [15].

These findings highlight the importance of performing diagnostic tests in different positions when a patient presents with positional changes in dyspnea. By altering the patient’s position during the examination, the underlying cause of POS may be more readily identified, leading to an accurate diagnosis and appropriate treatment [16].

## Conclusions

We report a case of POS caused by an ASD along with an atrial septal aneurysm and an inferior vena cava valve, which was diagnosed using both transthoracic echocardiography in the upright position and transesophageal echocardiography. The key messages from this report are the importance of carefully listening to the patient’s chief complaint of positional dyspnea that paradoxically improves with walking, which should raise suspicion for POS, and that when POS is suspected, performing diagnostic tests with positional changes may provide crucial information leading to the correct diagnosis.
